# Supplementary material for: Structure and antigenicity of divergent Henipavirus fusion glycoproteins
Source: Nat Commun. 2023 Jun 16;14:3577. doi: 10.1038/s41467-023-39278-8 (PMC10275869; doi:10.1038/s41467-023-39278-8)
Supplement: Supplementary file 1 — Supplementary Information [file 41467_2023_39278_MOESM1_ESM.pdf]

Structure and antigenicity of divergent Henipavirus fusion glycoproteins

Ariel Isaacs†[1], Yu Shang Low†[1], Kyle L. Macauslane[1], Joy Seitanidou[1], Cassandra L. Pegg[1], Stacey T.M. Cheung[1], Benjamin Liang[1], Connor A.P. Scott[1], Michael J. Landsberg[1,2], Benjamin L. Schulz[1,2], Keith J. Chappell[1,2,3], Naphak Modhiran*[1], Daniel Watterson*[1,2]

[1]School of Chemistry and Molecular Bioscience, The University of Queensland, Brisbane, Australia

[2] Australian Infectious Disease Research Centre, The University of Queensland, Brisbane, Australia

[3]Australian Institute for Bioengineering and Nanotechnology, Brisbane, Australia

† These authors contributed equally

* These authors jointly supervised this work

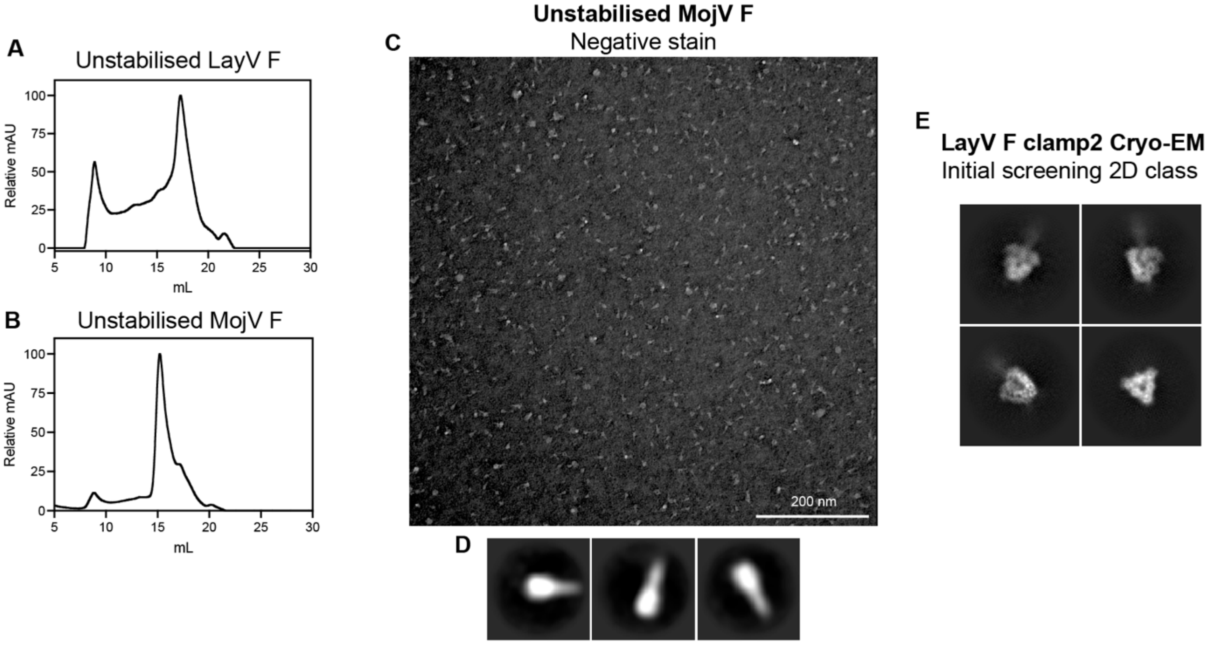

Figure S1 Size-exclusion chromatography of unstabilised LayV F (A) and MojV F (B). (C) Representative negative stain image (1 of 25 images independently acquired) of unstabilised MojV F trimer with 2D class averages shown in (D). (E) Initial screening cryo-EM 2D classes of LayV F clamp2 proteins. Source data are provided as a source data file.

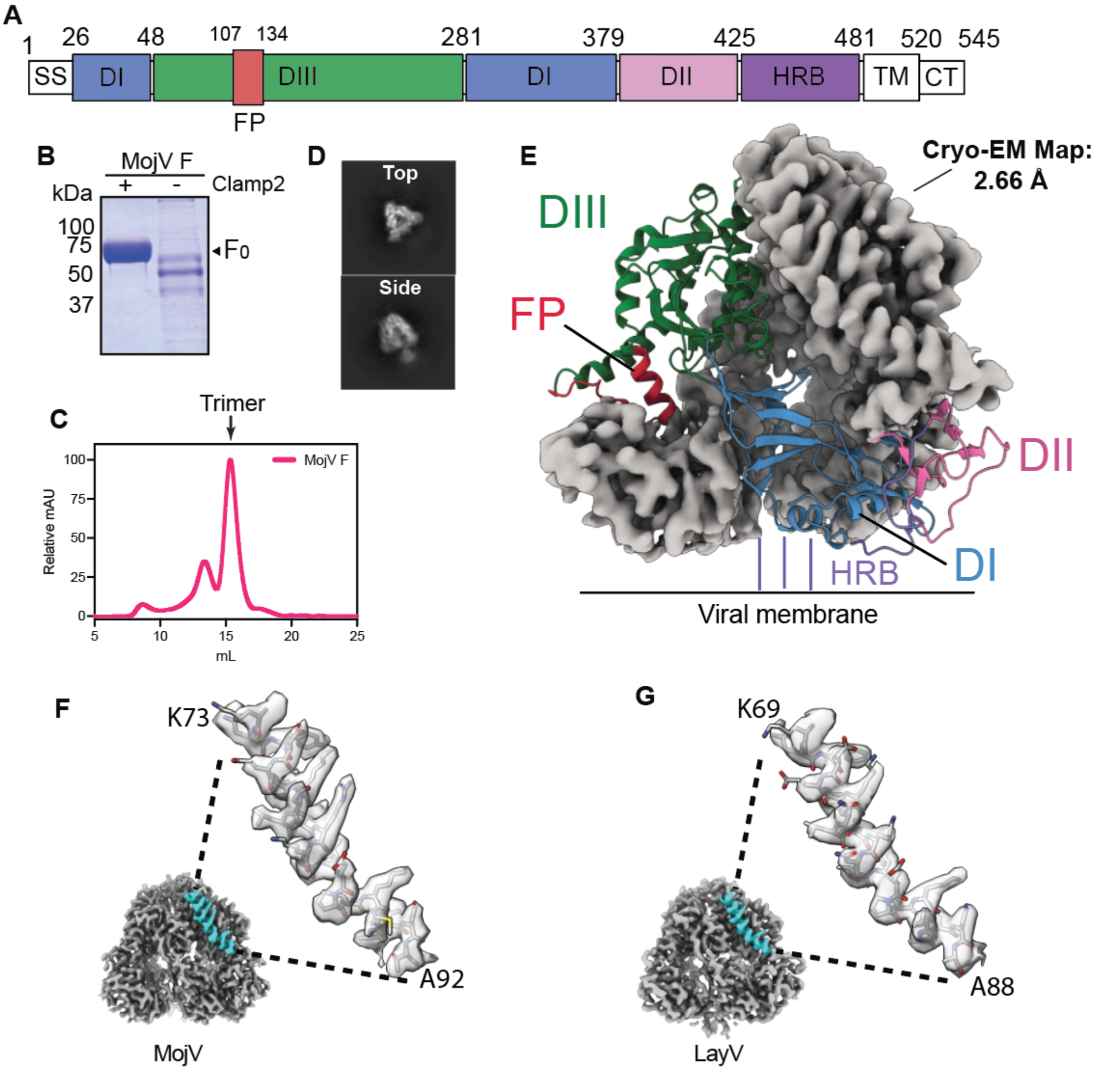

Figure S2 Cryo-EM structure of prefusion MojV F. (A) Gene schematic of MojV F with each domain coloured. Domains coloured in white are not included in the final structure. SDS-PAGE of purified MpjV F proteins under reducing conditions either stabilized with clamp2 or unstabilised. (C) SEC of MojV F stabilized with clamp2 or unstabilised ran on Superose 6 Increase 10/300 GL column. (D) Representative 2D class averages of prefusion stabilized MojV F. (E) Final cryo-EM structure and model solved to 2.66 Å with model domains is coloured as in (A). Map quality illustrated for the outwards facing surface exposed helix of DIII for MojV (F) and LayV (G). Source data are provided as a source data file.

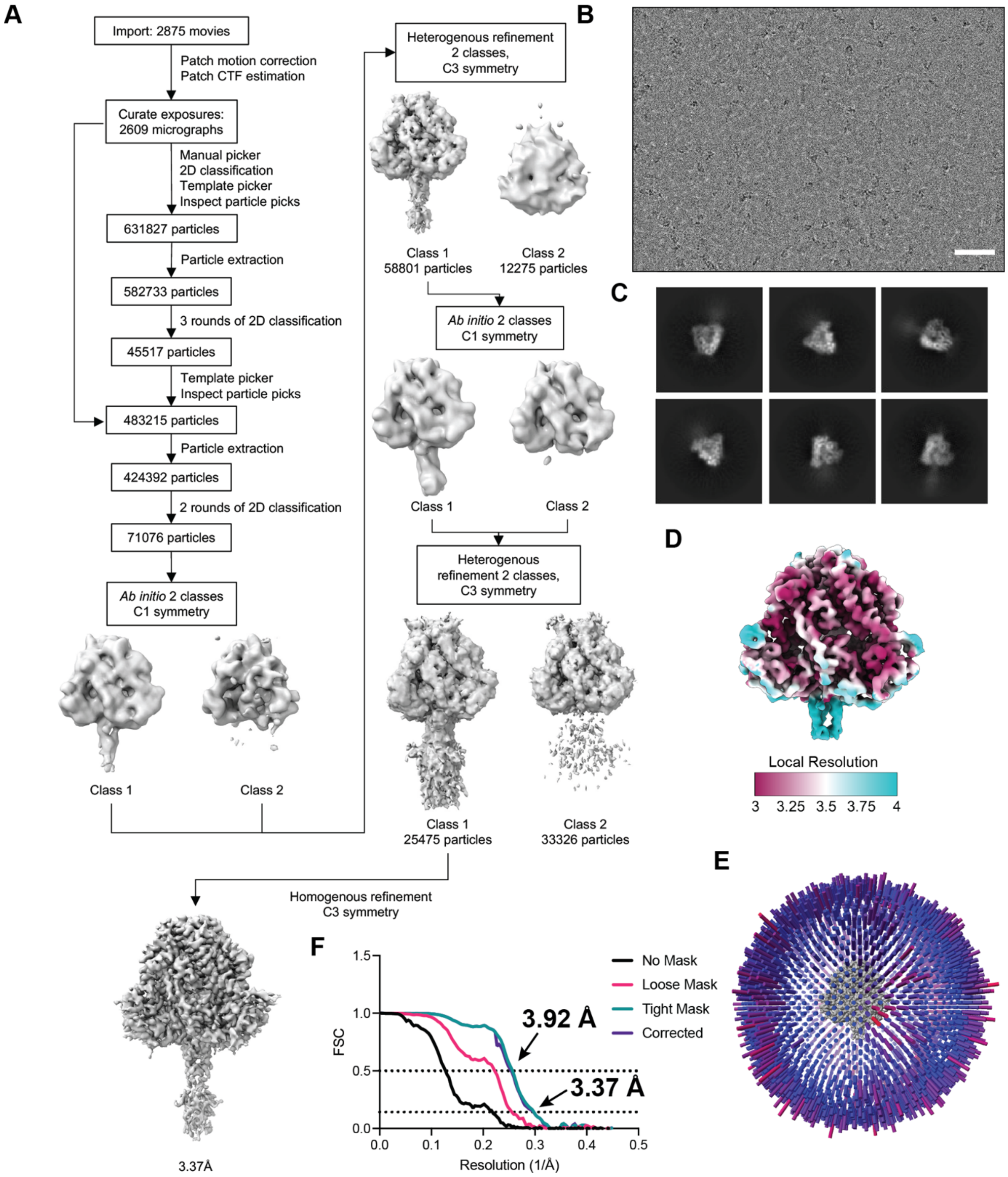

Figure S3 (A) Cryo-EM data acquisition and workflow on cryoSPARC for LayV F. Final structure at 3.37 Å. (B) Representative micrograph for LayV F. Scale bar shown is 50 nm. (C) Representative cryo-EM 2D class averages. (D) Local resolution map of LayV F generated by cryoSPRAC. (E) Angular distribution of particles used in final reconstruction. (F) Fourier shell correlation (FSC) curve of final map. Dotted lines show cutoffs at 0.5 and 0.143.

	**Langya virus F protein (PDB ID: 8FMX, EMDB-29299)**	**Mojiang virus F protein (PDB ID: 8FMY, EMD-29300)**
Data collection and processing		
Magnification	60,000x	100,000x
Voltage (kV)	300	300
Electron exposure (e– $Å^{-2}$)	40	40
Defocus range (μm)	−0.5 to −2.5	−0.5 to −2.5
Pixel size (Å)	0.4 (super resolution)	0.25 (super resolution)
Symmetry imposed	C3	C3
Initial particle images (no.)	424,392	2,054,371
Final particle images (no.)	25,475	213,754
Map resolution (Å)	3.37	2.66
FSC threshold	0.143	0.143
Map resolution range (Å)	2.4 – 6.2	2.45 – 3.8
Refinement		
Initial model used (PDB code)	n/a (ModelAngelo)	n/a (ModelAngelo)
Model resolution (Å)	3.35	2.66
FSC threshold	0.143	0.143
Map sharpening B factor ($Å^2$)	-88.4	-102.7
Model composition		
Non-hydrogen atoms	10329	9597
Protein residues	1335	1260
Ligands	12	0
R.m.s. deviations		
Bond lengths (Å)	0.004 (0)	0.008 (0)
Bond angles (°)	0.903 (7)	1.159 (12)
Validation		
MolProbity score	0.78	0.65
Clashscore	0.43	0.31
Poor rotamers (%)	0	0
Ramachandran plot		
Favored (%)	97.37	97.85
Allowed (%)	2.63	2.15
Disallowed (%)	0	0
Rama-Z (No. of residues analysed, Ramachandran plot Z-score, RMSD)		
Whole	N = 1329, -1.06 (0.21)	N = 1254, -1.16 (0.21)
Helix	N= 453, -0.60 (0.22)	N = 402, -1.03 (0.21)
Sheet	N = 261, -0.18 (0.32)	N = 258, 0.31 (0.31)
Loop	N = 615, -0.89 (0.23)	N = 594, -0.98 (0.23)

Figure S4 – Model generation and cryo-EM data acquisition details for MojV and LayV F proteins

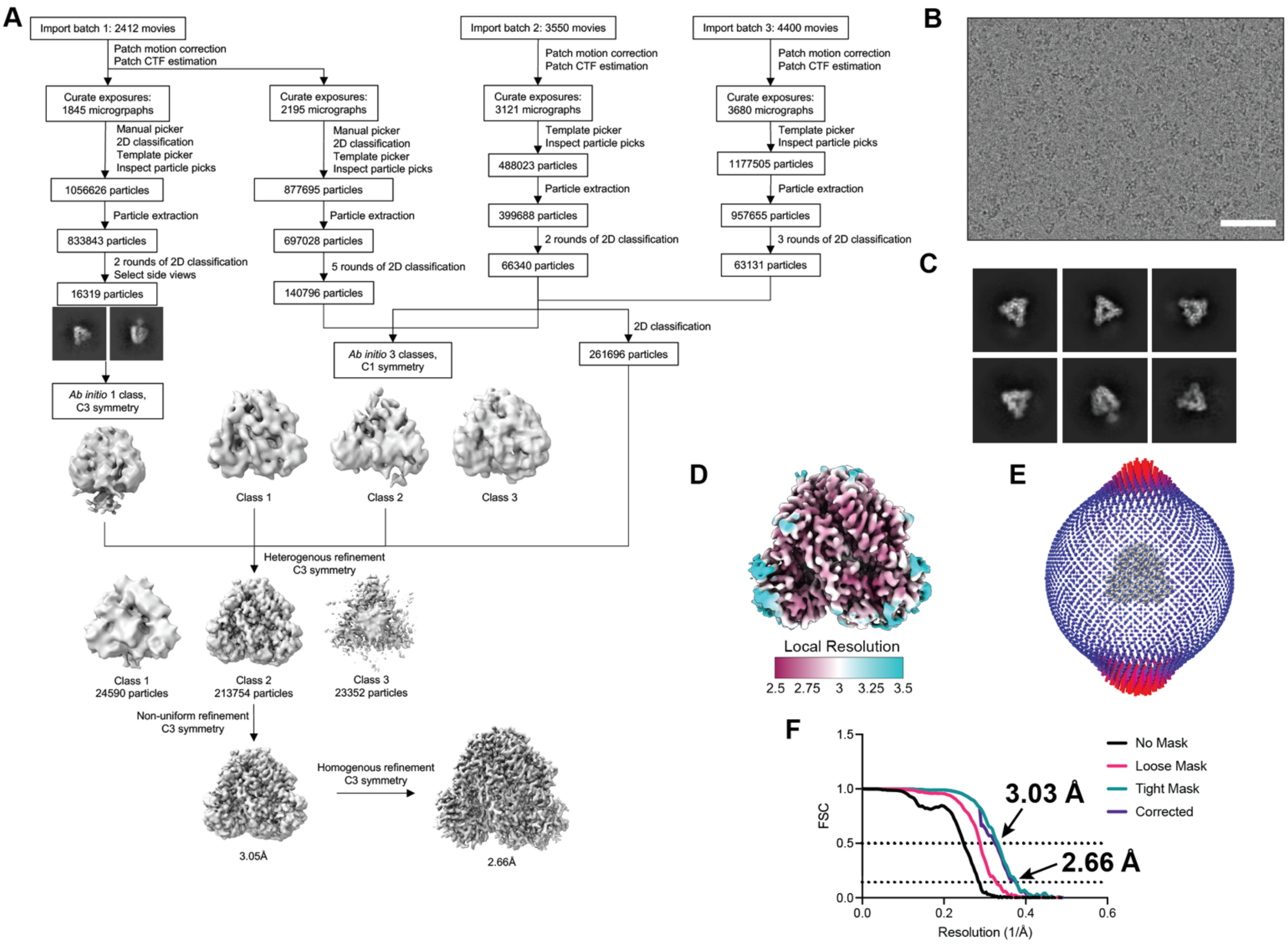

Figure S5 (A) Cryo-EM data acquisition and workflow on cryoSPARC for MojV F. Final structure at 2.66 Å. (B) Representative micrograph for MojV F. Scale bar shown is 50 nm. (C) Representative 2D class averages. (D) Local resolution map of MojV F generated by cryoSPRAC. (E) Angular distribution of particles used in final reconstruction. (F) Fourier shell correlation (FSC) curve of final map. Dotted lines show cutoffs at 0.5 and 0.143.

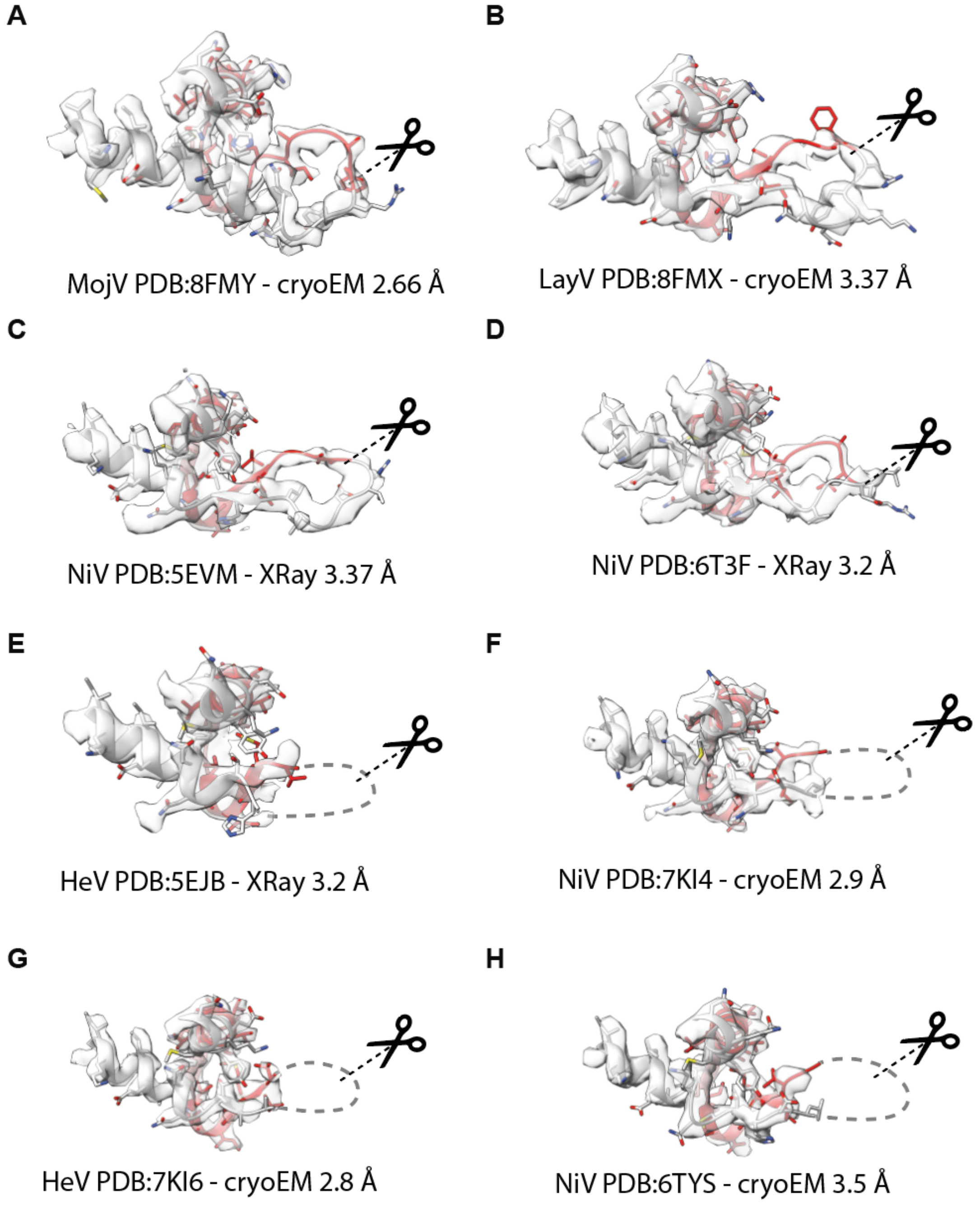

Figure S6 Comparison of the F1/F2 cleavage site and fusion loop regions for published henipavirus prefusion F structures. Despite high sequence homology, MojV (A) and LayV (B) adopt different conformations. LayV site is extended and similar to the publishes crystal structures of Apo NiV F (C) and mAb66 bound NiV F (D). The surface exposed cleavage site was not resolved for HeV F (E), or in other antibody complexed NiV structures (F-H).

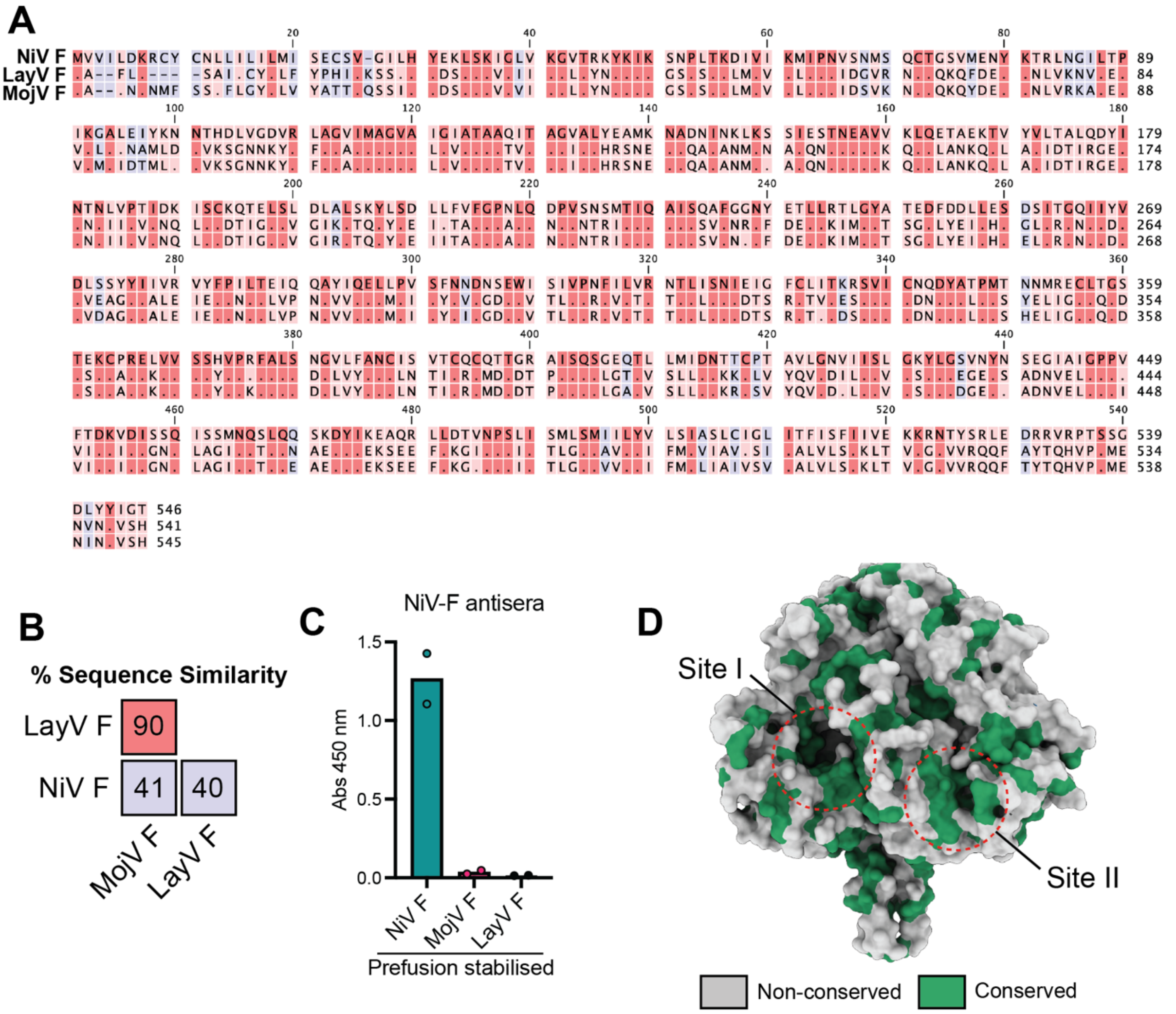

Figure S7 - (A) Sequence alignment between NiV, MojV and LayV F proteins. (B) Pairwise comparison of full-length F amino acids generated on CLC Workbench. (C) NiV F antisera reactivity against prefusion stabilized F proteins. Reactivity conducted by indirect ELISA with a 1:20 dilution of sera (n = 2). (D) Surface representation structural alignment of LayV and NiV F proteins with conserved regions shown in green and non-conserved shown in grey. Two conserved pockets are depicted as site I and II. Source data are provided as a source data file.
