# Supplementary material for: Structure and antigenicity of divergent Henipavirus fusion glycoproteins
Source: Nat Commun. 2023 Jun 16;14:3577. doi: 10.1038/s41467-023-39278-8 (PMC10275869; doi:10.1038/s41467-023-39278-8)
Supplement: Supplementary file 2 — Reporting Summary [file 41467_2023_39278_MOESM2_ESM.pdf]

nature portfolio

Corresponding author(s): Naphak Modhiran, Daniel Watterson

Last updated by author(s): May 3, 2023

Reporting Summary

Nature Portfolio wishes to improve the reproducibility of the work that we publish. This form provides structure for consistency and transparency in reporting. For further information on Nature Portfolio policies, see our Editorial Policies and the Editorial Policy Checklist.

Statistics

For all statistical analyses, confirm that the following items are present in the figure legend, table legend, main text, or Methods section.

n/a	Confirmed	
☐	☒	The exact sample size (*n*) for each experimental group/condition, given as a discrete number and unit of measurement
☒	☐	A statement on whether measurements were taken from distinct samples or whether the same sample was measured repeatedly
☒	☐	The statistical test(s) used AND whether they are one- or two-sided<br>*Only common tests should be described solely by name; describe more complex techniques in the Methods section.*
☒	☐	A description of all covariates tested
☒	☐	A description of any assumptions or corrections, such as tests of normality and adjustment for multiple comparisons
☐	☒	A full description of the statistical parameters including central tendency (e.g. means) or other basic estimates (e.g. regression coefficient) AND variation (e.g. standard deviation) or associated estimates of uncertainty (e.g. confidence intervals)
☒	☐	For null hypothesis testing, the test statistic (e.g. *F*, *t*, *r*) with confidence intervals, effect sizes, degrees of freedom and *P* value noted<br>*Give P values as exact values whenever suitable.*
☒	☐	For Bayesian analysis, information on the choice of priors and Markov chain Monte Carlo settings
☒	☐	For hierarchical and complex designs, identification of the appropriate level for tests and full reporting of outcomes
☒	☐	Estimates of effect sizes (e.g. Cohen's *d*, Pearson's *r*), indicating how they were calculated

Our web collection on statistics for biologists contains articles on many of the points above.

Software and code

Policy information about availability of computer code

Data collection	SerialEM software v.3.1 and yoneoLocr
Data analysis	cryoSPARC v.3.3.1; ModelAngelo v.0.2.2; Coot v.0.9.8.1; Chimera v.1.17; ChimeraX v.1.3; Phenix v.1.20.1-4887; Byonic v.4.3.4; GraphPad Prism v.9.4.1

For manuscripts utilizing custom algorithms or software that are central to the research but not yet described in published literature, software must be made available to editors and reviewers. We strongly encourage code deposition in a community repository (e.g. GitHub). See the Nature Portfolio guidelines for submitting code & software for further information.

Data

Policy information about availability of data

All manuscripts must include a data availability statement. This statement should provide the following information, where applicable:

- Accession codes, unique identifiers, or web links for publicly available datasets
- A description of any restrictions on data availability
- For clinical datasets or third party data, please ensure that the statement adheres to our policy

The cryo-EM data generated in this study have been deposited in the Electron Microscopy Data Bank & Protein Data Bank databases under accession codes EMDB-29299 [https://www.ebi.ac.uk/pdbe/entry/emdb/EMD-29299]/PDB 8FMX [https://doi.org/10.2210/pdb8FMX/pdb] (LayV F) and EMDB-29300 [https://www.ebi.ac.uk/pdbe/entry/emdb/EMD-29300]/PDB 8FMY[https://doi.org/10.2210/pdb8FMY/pdb] (MojV F). The processed mass spectrometry glycoproteomics

data are available at ProteomeXchange Consortium via the PRIDE partner repository with the dataset identifier PXD039898 [https://www.ebi.ac.uk/pride/archive/projects/PXD039898]. The source data generated in this study are provided in the Source Data file. The Nipah antibody data used in this study are available in the Protein Data Bank database under accession codes 6TYS [https://doi.org/10.2210/pdb6TYS/pdb] (5B3), 6T3F [https://doi.org/10.2210/pdb6T3F/pdb] (mAb66), 7KI4 [https://doi.org/10.2210/pdb7KI4/pdb] (12B2) and 7KI6 [https://doi.org/10.2210/pdb7KI6/pdb] (1F5). The data that support the findings of this study are available from the corresponding author upon request with a Materials Transfer Agreement.

Human research participants

Policy information about studies involving human research participants and Sex and Gender in Research.

Reporting on sex and gender	NA
Population characteristics	NA
Recruitment	NA
Ethics oversight	NA

Note that full information on the approval of the study protocol must also be provided in the manuscript.

Field-specific reporting

Please select the one below that is the best fit for your research. If you are not sure, read the appropriate sections before making your selection.

☒ Life sciences ☐ Behavioural & social sciences ☐ Ecological, evolutionary & environmental sciences

For a reference copy of the document with all sections, see nature.com/documents/nr-reporting-summary-flat.pdf

Life sciences study design

All studies must disclose on these points even when the disclosure is negative.

Sample size	Cryo-EM sample size was not predetermined. Each cryo-EM dataset contained thousands of particles. The size of each dataset was based on instrument availability. Relevant particle numbers for each dataset are provided in Fig S3 & S5. For ELISA, two replicates were included and were sufficient as no quantitative affinity measures were deduced.
Data exclusions	Cryo-EM images of poor quality were removed based on assessment of the contrast transfer function and resolution in 2D and 3D. LayV F particles with missing stem density were also removed to achieve a more complete structure. Please refer to workflow in Fig S3.
Replication	Cryo-EM data collection was performed once for LayV F sample (Fig S3) and three times for MojV F sample (Fig S5). Fig 3b-e, two individual datapoints are shown representing mean of replicates. Fig S7c, two individual data points are shown representing mean of replicates.
Randomization	Cryo-EM data were randomised into two half-datasets and independently refined. Fourier Shell Correlation between two half-datasets was used to provide a "gold-standard" estimate of the resolution of the cryo-EM map. This is provided in Fig S3 and S5.
Blinding	Blinding is not relevant. Analysis and collection of cryo-EM data did not require statistical interpretation and human bias is mitigated by established validation metrics.

Reporting for specific materials, systems and methods

We require information from authors about some types of materials, experimental systems and methods used in many studies. Here, indicate whether each material, system or method listed is relevant to your study. If you are not sure if a list item applies to your research, read the appropriate section before selecting a response.

Materials & experimental systems

n/a	Involved in the study	
☐	☒	Antibodies
☐	☒	Eukaryotic cell lines
☒	☐	Palaeontology and archaeology
☒	☐	Animals and other organisms
☒	☐	Clinical data
☒	☐	Dual use research of concern

Methods

n/a	Involved in the study	
☒	☐	ChIP-seq
☒	☐	Flow cytometry
☒	☐	MRI-based neuroimaging

Antibodies

Antibodies used	Nipah F specific antibodies (5B3, mAb66, 12B2, 1F5) were made in-house. Goat anti-human HRP antibody (Invitrogen, Lot 58-47-090117).
Validation	Validation for binding against NiV F provided in this publication for specificity against NiV F and species by reactivity with anti-human secondary (Fig 3b-e). Validation of these mAbs also provided by original work:<br>5B3- Dang, H.V., Cross, R.W., Borisevich, V. et al. Broadly neutralizing antibody cocktails targeting Nipah virus and Hendra virus fusion glycoproteins. Nat Struct Mol Biol 28, 426–434 (2021). https://doi.org/10.1038/s41594-021-00584-8<br>12B2 & 1F5 - Dang, H.V., Cross, R.W., Borisevich, V. et al. Broadly neutralizing antibody cocktails targeting Nipah virus and Hendra virus fusion glycoproteins. Nat Struct Mol Biol 28, 426–434 (2021). https://doi.org/10.1038/s41594-021-00584-8<br>mAb66 -Avanzato V.A., Oguntuyo K.Y., Escalera-Zamudio M., et al. A structural basis for antibody-mediated neutralization of Nipah virus reveals a site of vulnerability at the fusion glycoprotein apex. Proc Natl Acad Sci U S A. (2019) Dec 10;116(50):25057-25067. doi: 10.1073/pnas.1912503116<br>Other mAbs used:<br>Goat anti-human HRP secondary: https://www.thermofisher.com/antibody/product/Goat-anti-Human-IgG-Fc-Highly-Cross-Adsorbed-Secondary-Antibody-Polyclonal/A18829

Eukaryotic cell lines

Policy information about cell lines and Sex and Gender in Research

Cell line source(s)	ExpiCHO-S (ThermoFisher)
Authentication	Not authenticated
Mycoplasma contamination	Not tested
Commonly misidentified lines<br>(See ICLAC register)	No commonly misidentified cell lines used.
